# Measuring participation for persons with mental illness: A systematic review assessing relevance of existing scales for low and middle income countries

**DOI:** 10.1186/s40359-015-0093-0

**Published:** 2015-10-14

**Authors:** Ganesh M. Babulal, Parul Bakhshi, Sunyata Kopriva, Sarah A. Ali, Susan A. Goette, Jean-Francois Trani

**Affiliations:** Department of Neurology, Washington University School of Medicine, Campus Box 8111, 4488 Forest Park, St. Louis, MO 63110 USA; Program in Occupational Therapy, Washington University School of Medicine, St. Louis, MO USA; Brown School of Social Work, Washington University, St. Louis, MO USA; Cultural Resources Consulting, Minneapolis, MN USA

**Keywords:** Participation, Mental illness, Low and middle income countries, Outcomes

## Abstract

**Background:**

Participation is increasingly becoming an important outcome for assessment in many fields, including development, disability and policy implementation. However, selecting specific instruments to measure participation has been a significant problem due to overlapping conceptual definitions and use of different theories. The objective of this paper is to identify participation instruments, examine theories/definition supporting their use and highlight scales for use in low and middle-income countries for persons with mental illness.

**Methods:**

A systematic literature review was conducted to identify instruments intended to measure participation for individuals with severe mental illness. The search was limited to peer-reviewed articles published in English between 2003 and 2014. Instruments that measured related concepts of well-being, quality of life and social functioning were also identified and screened for items that pertained to participation, defined as empowerment and collective capabilities.

**Results:**

Five scales met established criteria for assessing participation and were determined to contain questions measuring empowerment and/or collective capabilities. However, each scale largely assessed individual functioning and capacity, while neglecting collective aspects of the community. All scales were developed in high-income countries and none were used in low and middle-income countries.

**Conclusions:**

There is an urgent need for participation scales to focus on empowerment as well as collective capabilities. Further, development of participation scales should clearly delineate theoretical foundations and concepts used. Finally, participation scales used in low and middle income countries should consider how contextual factors like medicine, poverty and disability, particularly with regards to mental illness, impact content of the scale.

**Electronic supplementary material:**

The online version of this article (doi:10.1186/s40359-015-0093-0) contains supplementary material, which is available to authorized users.

## Background

The literature on poverty and disability in low and middle-income countries (LMICs) is growing [1–4] but little has been done to examine the association between mental illness, lack of participation resulting from stigma-related processes, and poverty. The mental health literature shows that persons with mental illness in LMICs are among the poorest [5]. In 11 community-based studies conducted in developing-country, significant associations between poverty indicators and common mental disorders were found in all but one study [6]. The literature also demonstrates that persons with mental illness consistently face what Corrigan and Watson [9] call public stigma; stereotypes adopted by a community regarding a specific group and related action against members of the target group through psychosocial processes that result in exclusion [7, 8]. Stereotypes of mental illness are widespread in many societies and “include dangerousness, incompetence, and character weakness” (Corrigan and Watson, [9]: 181). Such negative stereotypes often trigger prejudicial attitudes, which may result in a specific behavior of discrimination such as refusing to hire a person with mental illness or keeping them indoors and away from public view. Stigma-related processes reduce social participation and may worsen the situation of persons with mental illness by excluding them from the labor market.

This paper adopts the premise that persons with mental illness undergo limited participation in family, community and society at large, as a result of stigma-related processes. A better understanding of illness and of the existing social response may establish social factors shaping the prognosis of severe mental illness. This would offer newer avenues for public health interventions to complement biomedical treatment in LMICs [10]. However, it is crucial to grasp how participation is defined. In order to do this, there is an urgent need to pinpoint the concept of participation and identify culturally appropriate measures of individual participation.

In the first section of this paper, we discuss “participation” as a concept and propose the capabilities approach (CA) as a framework for delimiting the term. In the second section, we present the implications of the medical view of participation as a health outcome. In the third section, we detail the methods we used in our systematic review of the literature on participation measures in LMICs. In the fourth section, we present the findings from the review and discuss its implications.

### Participation as theory and concept in LMICs

In the field of development, participation as a concept gained momentum through adoption and use in academic institutions, local governments and international organizations like the World Bank [11, 12]. Participation emerged as a suitable concept for use in mainstream issues of empowerment and ownership of policies and interventions by the beneficiaries of development [13], by giving a voice and a role to the poor and marginalized individuals in decisions making processes. In practice, the absence of well-defined principles to operationalize participation in LMICs has yielded poor outcomes. The World Bank and the International Monetary Fund attempts to discuss poverty reduction strategic papers (PRSP) in order to enhance domestic accountability have yielded unsatisfactory results in terms of ensuring participation and ownership of vulnerable groups [14–16].

One of the central issues is that participation can be defined at various levels. Arnstein [17] suggested classifying participation in eight levels across domains of nonparticipation, tokenism and citizen power. In this typology, at the highest level, citizens or ‘actors’ who have power are able to structure policies and programs; at the lowest level, participation is synonymous with consultation or information to maintain the status quo. Pretty [18] proposed a similar typology depicting a spectrum of power shifts from authorities to regular persons. In this view, participation is conceptualized as opportunities for the poor and vulnerable individuals, those without bargaining power, to express a voice and gain some benefits in social interactions with other more privileged individuals in society [19]. In practice, these opportunities for participation are overlooked by many development agencies (United Nations institutions such as the World Bank, International Non Governmental Organizations) and limited to mere consultation. This disconnect of where the term “participation” can be used to describe a variety of processes (that may or may not question power dynamics) explains why many development interventions fail to address economic inequalities and social injustice resulting from the current globalization process [11, 20].

Despite countless critiques [15, 16, 21, 22], participation remains a central principle in the field of development to enhance development effectiveness following the 2005 Paris Declaration on Aid Effectiveness [23]. Advocates argue that participation still has a strong contribution to make on conditions that it is carefully appraised by relinking it to its ideological origins of social transformation and empowerment of the poor and vulnerable [13]. We argue that the capability approach (CA) as a specific framework can be useful in order to rethink development outcomes by focusing on the enhancement of individual well-being understood as expansion of individual capabilities and choices. Within this perspective, human development consists of expanding valuable freedoms where a “set of capabilities” is defined as functionings among opportunities that an individual chooses [24]. The freedom to exercise chosen functionings, doings and beings by an individual is a central dimension of quality of life in the CA [25]. As a result, the CA has been proposed as a framework for quality of life measurement [26, 27]. Within the CA, basic capabilities needed to escape poverty should at the very minimum include the freedom to be healthy, to be educated, to be well nourished, to be well sheltered, freedom to live peaceful lives away from violence, freedom to appear in public without shame and freedom to participate in the community life among others [28]. The concept of “agency” constitutes the capacity of individuals to see themselves as the main decision-makers with the ability to make choices for her/his life. An agent is “*someone who acts and brings about change*” and is there is empowered to take action (Sen [24], p19). Furthermore, agency embodies the ability of an individual to 1) individually and collectively engage in processes that can lead to social transformation, 2) question the power dynamics that contribute to inequality and 3) improve the well-being of both individuals and the community as a whole [29–31]. Participation in collective action by exercising collective agency, can lead to the expansion of the capability set for the participating members of the group who take action together to secure the expansion of the collective capabilities set [30, 32, 33]. Despite focusing on the individual, the CA recognizes the social space wherein choices are determined [34]. Stewart & Deneulin state that “*flourishing individuals generally need and depend on functional families, cooperative and high-trust societies, and social contexts which contribute to the development of individuals who choose“valuable” capabilities*” (2002, p. 68). Based on human development and the capabilities approach, there is an emphasis on the power of the person to individually and collectively change the social order through her/his “participation” in the process of social transformation. While participation has been essentially linked to development theory and practice in LMICs, it has been used as a concept to measure individual function in high-income countries (HICs).

### Participation as a health outcome in high-income countries

Scale development and validation is based on a clearly defined concept with explicit references to theory or philosophy. In medical practice and research, participation as a measurement outcome in HICs has been linked to the International Classification of Functioning, Disability, and Health (ICF) [35]. The ICF defines participation as “involvement in a life situation” (p.10); this definition has been rapidly adopted and used as a measurement outcome of health [36–38]. However, adoption of the ICF’s participation has produced a myriad of problems with measuring participation [39]. Without a historical premise, a philosophical description, or a theoretical grounding to validate linkages to health, participation is challenging to operationalize [40]. Other critiques include omitting the subjective aspect of meaning, choice and control, not accounting for the experience of persons with a disability and having conflated definitions with other ICF components like capacity, functioning and performance [41–43]. Additionally, literature reviews and meta-analyses have discussed significant overlap between participation and similar concepts like well-being, quality of life, activity, social performance and general physical functioning [44–46]. Yet, despite challenges with an obfuscated definition and conflation with other health-related outcomes, participation continues to be used as a terminal outcome for health. Based on the authors’ experience, participation instruments developed in HICs based on the ICF are being used and modified in LMICs with a range of different cultural settings. However, this practice runs the risk of producing flawed data and a biased estimate of participation since the initial context and purpose of the scale has changed and cultural adaptation can be challenging [47]. A critique of this practice is that participation is viewed in HICs as individualistic functioning separate from collective interaction, while in LMICs, the focus of participation is on engagement of people in society, as well as the collective responsibility to allow for such engagement [46]. A common understanding for measuring participation suggests incorporating engagement of both the individual and the collective in a society. Trani et al. [48] have argued using the example of persons with disability in LMICs that the CA, by focusing on agency, goes beyond the ICF by looking at individuals’ choices, beliefs and preferences within a given economic, social and cultural environment able to provide opportunities for or to create barriers to human development. Yet, in the field of psychiatry, participation of individuals with a mental condition does not encompass agency but is restricted to measuring social functioning. Specific instruments have been used in this field to measure social functioning defined as involvement with other individuals in various social situations: social engagement and communication with peers, intimate relationships, social behavior and skills at home, in recreational activity, at school or at work but without exploring the meaning and subjective experience of these interactions [49–52].

### Study aims

This paper has several aims. 1) Identify measures or instruments in a systematic review that evaluate participation and related concepts like quality of life, well-being or social functioning. 2) Evaluate whether the identified measures and instruments assess participation in the context of the capability approach. 3) Highlight scales that may be relevant to evaluating participation of persons with mental illness in LMICs.

In order to concretely identify the scales and measurements that would be contextually relevant for assessing participation in LMICs, we apply a strict definition of what constitutes “participation”, building on concepts discussed above. More precisely, we analyze the documents screened through the review according to two criteria. First, we look for tools that define participation as empowerment or agency, following the CA. Second, we identified tools that assess collective capabilities, going beyond individual functioning, experience or opinion and providing insight on achievements of the collective unit (family, community) or an understanding of the social and cultural context.

## Methods

A literature review was conducted to identify instruments intended to measure participation for individuals with severe mental illness (schizophrenia, manic depression, bipolar, etc.). Instruments that measured related concepts of well-being, quality of life and social functioning were also identified and screened for items that pertained to participation, defined as empowerment and collective capabilities. The search was limited to peer-reviewed articles published in English between 2003 and 2014. Specific mesh terms were established and used in selected databases. Final article inclusion criteria following abstract review were: date of publication (2003–2014) and reference to an instrument used to measure one of the four mental health constructs being examined (participation, well-being, quality of life or social functioning).

The following databases were searched first: Mental Health Measurements Yearbook, PsychARTICLES, PsychInfo, PsychTESTS, SocINDEX, Global Health, CINAHL Plus, MEDLINE. The search strategy for all databases, except Mental Health Measurements Yearbook, had three different components. The first two components remained constant.

The fist component was [“psychometric*” OR “measure*” OR “scale*” OR “index” OR “instrument*” OR “test*” OR “tool*”]. The second component was [“mental health” OR “mental* ill*” OR “mental disease*” OR “mental disorder*” OR “mental problem*” OR “mental issues” OR “psychologic* health” OR “psychologic* ill*” OR “psychologic* disease*” OR “psychologic* disorder*” OR “psychologic* problem” OR “psychologic* issue*” OR “psychiatric health” OR “Psychiatric ill*” OR “psychiatric disease*” OR “psychiatric disorder*” OR “psychiatric problem*” OR “psychiatric issues” OR “depression” OR “major depression” OR “major depress* disorder*” OR “severe depression” OR “mood disorder*” OR “severe mood disorder*” OR “severe affective disorder” OR “affective disorder*” OR “psycho-social disorder*” OR “severe psycho-social disorder*” OR “schizophrenia” OR “bipolar depression” OR “severe mental* ill*” OR “severe mental disorder*”]. The third component involved the specific mental health variables: well-being, quality of life, participation and social functioning. The mesh terms for the third component were as follows: [“well-being” OR “well being” OR “wellbeing”], [“quality of life”], [“participation”] and [“social functioning”]. The three components were searched individually and then together. A complete final search would be: component 1 AND component 2 AND one component three variable. Mental Health Measurements Yearbook search strategy only included component 3, because of the nature of the database. Once duplicates were removed in RefWorks, the results were then exported into EPPI-Reviewer 4 and screened on the titles and abstract. Articles were excluded on the following criteria: (1) published before 2003, (2) no reference or mention of scale tool, instrument or measurement, (3) no reference to participation, quality of life, well-being or social functioning or (4) article was a commentary note, book chapter, manual or non-peer-reviewed paper. Articles remaining after the initial screening were reviewed using the keywording tool. The keywording tool included: (1) construct being measured (participation, well-being, quality of life, social functioning), (2) phenomenon measured (mental health, physical health, other), (3) population profile (country of focus, type of population, source of issue, age, gender), (4) study type (review, qualitative quantitative, mixed methods, other, not specified), (5) name of scale and (6) standardized in an LMIC (Additional file 1).

## Results

Instruments that were created for, or have been used in, LMICs were given special consideration. However, articles from high-income countries were also reviewed, because of the proliferation of mental health research in these countries. Following the outlined search strategies, 191 abstracts met initial inclusion criteria and were imported for review. After title and abstracts were reviewed for duplicates, 143 remained and were exported into EPPI-Reviewer 4. After screening on title and abstract, 85 articles met inclusion criteria and were reviewed to identify the scale that they referred to. There were 48 scales identified using the key wording tool; 14 of which were unavailable from Internet searches and the authors of the scale or needed to be purchased. The remaining 34 scales were obtained through open access and evaluated with the participation definition as outlined earlier. The identification of relevant items was done independently by two of the authors. Any disagreements were discussed and clarified with the rest of the authors. Five scales met the criteria as defined as having elements of participation. Table 1 lists the scales and describes the number of items in the scale, key items that measure empowerment and/or collective capabilities, underlying theory or conceptual definition, and use in LMICs. The original papers discussing the development and psychometric properties of the selected scales were obtained and reviewed to comment on scope of items in the instrument, theory or conceptual definition used and validation in LMICs.

**Table 1:** Scales meeting criteria of analyses

Scale	Total items Length (mins)	Key items that measure empowerment	Theory or conceptual definition	Articles identified in present review using scale
	**Used in LMIC’s**	**Key items that measure collective capability/ agency**		
Self-efficacy for social participation	27 15 mins	4. I feel I am a valuable person in society. 7. I can keep up with the changes in society. 18. If I try, I have the power to change society.	Bandura’s Self Efficacy Empowerment Social participation Social integration	Amagai, M., Suzuki, M., Shibata, F., & Tsai, J. (2012). Development of an instrument to measure self-efficacy for social participation of people with mental illness. *Archives of psychiatric nursing*,*26* (3), 240–248.
	No	20. There are people who accept me. 23. Others believe in my recovery.		
Assessment of quality of life	15 10 mins	None	Health related quality of life theoretical model WHO model of impairment and disability (ICIDH)	Atlantis, E., Goldney, R. D., Eckert, K. A., & Taylor, A. W. (2012). Trends in health-related quality of life and health service use associated with body mass index and comorbid major depression in South Australia, 1998–2008. *Quality of Life Research*, *21* (10), 1695–1704.
	No	9. Thinking about my health and my relationship with my family: A. My role in the family is unaffected by my health. B. There are some parts of my family role I cannot carry out. C. There are many parts of my family role I cannot carry out. D. I cannot carry out any part of my family role.		
Schizophrenia quality of life questionnaire	18 10 mins	5. I feel free to make decisions. 6. I feel free to act.	Health related quality of life	Baumstarck, K., Boyer, L., Boucekine, M., Aghababian, V., Parola, N., Lançon, C., & Auquier, P. (2013). Self-reported quality of life measure is reliable and valid in adult patients suffering from schizophrenia with executive impairment. *Schizophrenia research*, *147* (1), 58–67.
	No	10. I am helped and supported by my family. 11. My family pays attention to me 12. I am helped and supported by my friends or my relatives		
Lehman’s Quality of Life Interview	26 15 mins	None	Quality of life	Chávez, L. M., Canino, G., Negrón, G., Shrout, P. E., Matías-Carrelo, L. E., Aguilar-Gaxiola, S., & Hoppe, S. (2005). Psychometric properties of the Spanish version of two mental health outcome measures: World Health Organization Disability Assessment Schedule II and Lehman’s Quality of Life Interview. *Mental health services research*,*7* (3), 145–159.
	No	How do you feel about: 9. The way you and your family act toward each other? 10. The way things are in general between you and your family?		
International Classification of Functioning, Disability and Health	30 25 mins	None	WHO ICF	Tenorio-Martínez, R., del Carmen Lara-Muñoz, M., & Medina-Mora, M. E. (2009). Measurement of problems in activities and participation in patients with anxiety, depression and schizophrenia using the ICF checklist. *Social psychiatry and psychiatric epidemiology*, *44* (5), 377–384.
Checklist – Appendix 2				
	No	IV. Interpersonal Interactions (Capacity) (1) In your present state of health, how much difficulty do you have making new friends, without assistance? (2) How does this compare with someone, just like yourself only without your health condition? (Or: ”…than you had before you developed your health problem or had the accident?) (Performance) (1) In your present situation, how much of a problem do you actually have making friends? (2) Is this problem making friends made worse, or better, by anything (or anyone) in your surroundings? (3) Is your capacity to make friends, without assistance, more or less than what you actually do in your present surroundings? VI. Community, Social and Civic Life (Capacity) (1) In your present state of health, how much difficulty do you have participating in community gatherings, festivals or other local events, without assistance? (2) How does this compare with someone, just like yourself only without your health condition? (Or: ”…than you had before you developed your health problem or had the accident?) (Performance) (1) In your community, how much of a problem do you actually have participating in community gatherings, festivals or other local events? (2) Is this problem made worse, or better, by the way your community is arranged or the specially adapted tools, vehicles or whatever you use? (3) Is your capacity to participate in community events, without assistance, more or less than what you actually do in your present surroundings?		

### Self-efficacy for social participation (SESP)

The SESP, developed by Amagai et al. [53] in Japan is a condensed version of the original 37 items covering four dimensions: trust for social self, self-management, social adaptability and mutual support. The scale is intended for clinicians to use in helping to plan treatment for patients with mental illness in order to improve self-efficacy in social participation and community integration areas. We found three items (Table 1) that probe aspects of empowerment/agency and two items that probe on collective capabilities. In theory or conceptual definition, the authors directly cite Bandura’s theory of self-efficacy but do not cite sources when referencing empowerment, social participation and social integration [54]. The SESP is the only scale out of the five scales identified that contains items that moderately resonated with participation as outlined in this paper.

### Assessment of quality of life (AQoL)

The AQoL, developed by Hawthorne et al. [55] in Australia measures health-related quality of life (HRQoL) across five domains (illness, independent living, social relationships, physical senses, and psychological wellbeing). Hawthorne et al. developed their own theoretical model of HRQoL to support scale development and also referenced the WHO’s International Classification of Impairments, Disabilities, and Handicaps. The focus on HRQoL highlights the AQoL’s utility in many different health states. After reviewing the scale, one item (Table 1) was found to measure collective capabilities and none measured empowerment/agency.

### Schizophrenia quality of life questionnaire (S-QoL18)

The S-QoL18, developed by Auquier et al. [56] in France is a shortened version of the 41 item version designed to measure HRQoL in schizophrenia across eight domains (psychological well-being, self-esteem, family relationships, relationships with friends, resilience, physical well-being, autonomy and sentimental life). The S-QoL is designed to be completed by patients with schizophrenia to capture their perceptions and concerns; primary utility was intended to be in clinical trials as an outcome measure. The authors situate development of the S-QoL in the HRQoL literature but do not cite any theories or conceptual models. We found two items (Table 1) that attempt to measure empowerment/agency and three items that measure collective capabilities.

### Lehman’s quality of life interview (L-QoLI)

The L-QoLI, developed by Lehman [57] in France is a structured questionnaire that first obtains objective information on functioning and resources followed by subjective questions about the person’s satisfaction. The measure is largely used with patients who have psychiatric conditions to assess the social dimension of quality of life. The original 143 items scale has been reduced to 26 items across nine subscales in the subjective dimension (General quality of life, Living situation, Leisure, Family Relations, Social Relations, Finances, Work, Safety, and Health) and four subscales for the objective dimension (Leisure, Family Contacts, Social Contacts and Finances). The L-QoLI assesses overall quality of life but does not cite any underlying theory or conceptual definition. We found two questions (Table 1) that assess collective capabilities, and no question assessing empowerment/agency.

### International classification of functioning, disability and health (ICF Checklist)

The ICF Checklist, developed by the WHO in Switzerland is a questionnaire filled out by a health professional in clinical settings to assess a range of problems in order to determine the magnitude of disability for an individual. The checklist is based on the ICF model (WHO, [35]) and contains 125 categories across four domains: body functions, body structures, activities and participation, and environmental factors. The domain of activities and participation contains 47 categories with nine descriptions divided between capacity and performance. We found ten (Table 1) questions across the interpersonal interaction section and community, social and civic life section, which resonate with the collective aspect of participation.

## Discussion

Our findings show that very few scales include measures of participation in LMICs for persons with mental illness. Participation is a powerful concept in health, human development and social transformation; as such, its definition and measurement must be context-specific or risk difficulty with interpretation of its significance as an outcome. Defining participation in terms of empowerment/agency and ‘collective’ capabilities as an outcome supports the understanding of how social factors impact persons with mental illness in LMICs. Within our review, the SESP was the only scale identified that contained items which evaluated both empowerment and participation of the individual based on the capability approach. None of the five scales have been used in LMICs. More importantly, our review highlights a conflated definition of participation and a lack of measurement specificity of participation in mental health.

### Lack of a clear definition

The limited view of participation as individual functioning in mental health has led to ambiguous and overlapping definitions with concepts such as social functioning and quality of life. The intersection of participation, quality of life, well-being and social functioning obviates a clear understanding of (social) participation, which is further affected by a lack of theoretical or philosophical references. In the papers presenting scale development and validation, there were no clear or precise reference to theories; rather conceptual frameworks (e.g. ICF) and other concepts (e.g. HRQoL) were used for reference. Conceptual models and frameworks may be based on a theoretical concept but these are typically a constellation of relationship between different concepts to explain processes. Instead of being viewed as an empowering process based on a theory (i.e. the capability approach), participation in HICs has become synonymous with individual functioning and is measured through self-awareness of satisfaction or perceived quality of life. Participation is divorced from a collective, social world and does not account for power dynamics that contribute to barriers in participation. The added value of participation as a process that combines individual experiences of empowerment with enhancing of collective capabilities is also largely ignored in measurement.

### The need to move beyond the individual perspective in the field of mental illness

The corpus of existing measures from this review suggests an overt focus on the individual, specifically their awareness and perceptions but does not account for the collective nature of their problems. Most measures probe directly into the person’s satisfaction with different aspects of their life and do not strongly assess the impact on the family, friends or the immediate community. With the exception of the ICF Checklist, all of the scales were developed and validated in HICs, therefore underscoring how the ideology of participation is interpreted and perpetuated via these scales in LMICs. We found no scale with key items that strongly resonated with empowerment and collective capability/agency of persons with mental disorders or a scale that demonstrated a thorough understanding of participation via strong descriptions of theory.

We conducted a secondary review of the literature looking at participation in the context of other conditions than mental disorders. This search was performed using the databases Rehab Data and Web of Science, in order to explore data outside the discipline of mental health. The mesh terms outlined above in these databases yielded approximately 10,000 English articles published between 2003 and 2014. Mesh terms were narrowed to only include “participation” and “scale”. As a result, 898 articles were retrieved in Web of Science, and 264 in Rehab Data. After reviewing title and abstracts, over 100 scales were identified as measuring participation. Some of these scales not found in our original search included the Participation Scale [58], Assessments of Life Habits Scale [59], and Impact on Participation and Autonomy [60]. Our secondary search on “participation” and “measurement” in medicine yielded numerous scales outside of mental health literature in medicine that more strongly claim to measure participation as a process. Scales obtained in the secondary search appear to be underutilized in mental health/mental illness indicating marked differences in how participation is studied, applied and measured in medicine/rehabilitation and mental health. Our review brings up the question of why this gap exists and persists. To obtain a more thorough understanding of participation in mental health, future work should review these related scales, supporting theories or conceptual definitions and the types of context in which they are utilized.

### Choosing an instrument to measure participation of persons with mental illness in LMICs

The overall aim of this paper was to identify measures of participation, which aligned with the theory of participation as empowerment, social transformation and freedom of choice [24, 61, 62]. Such measures would be useful to evaluate participation of persons with mental illness in LMICs. In LMICs and even more so in conflict affected and fragile states, the health structures that address mental health of populations are at best weak and at worst non-existent [63]. As a result, persons with severe mental illness have no regular access to medication to manage their symptoms. Those with access to medications may not have a structured and informed healthcare system to support their mental illness. This poses difficulties for using individual-oriented scales to measure participation, social functioning or quality of life. As a result, the unit of analysis for determining positive outcomes needs to go beyond the individual and identify elements of collective dimensions of functioning by making spaces for perceptions of the family members and caregivers. Mental health care is not systematically viewed as a funding priority; as a result, assessments of living conditions need to not just gauge individual engagement but also identify the collective coping strategies that are in place and that need to be built upon to design an adequate and realistic response in terms of policy. Finally, solely individual-based scales need to be used with caution with extremely vulnerable and chronically poor populations due to the danger of obtaining results that reflect adaptive preferences resulting from habituation to prolonged and chronic deprivation and limited choices [64–67].

### Limitations

Similar to other systematic reviews, our limitations are primarily based on the search terms used. For example, ‘participation’ as a search term limited the number of studies found. Inclusion of related terms, such as ‘empowerment’, may have resulted in a greater number of scales for consideration. Further, since both searches were carried out in English and time period 2003–2014, it is likely possible that there are scales assessing participation in different languages and before 2003. Future studies will need to expand inclusion criteria to include different languages and longer time period.

## Conclusion

Our results suggest that existing measures of participation assessed individual functioning, capacity and performance. Philosophical and theoretical origins of participation are not clearly delimited in the development and validation of scales, thus leading to a clouded understanding of how the scale measures participation. The five scales identified in the search contain only a handful of items that reflect empowerment/agency and collective capabilities, and only one scale (SESP) contained items for both. The scant number of items in these measures highlights the need for expanding assessment of participation to include collective capabilities. Scales used to measure social exclusion also contain references to participation using the limited lens of performance in a collective setting (Baumgartner & Burns) [68]. It is evident that clearer theoretical foundations as well as discussion are required to re-define participation in the fields of development, medicine, poverty and disability, particularly with regards to mental illness.

The fact that there are few scales that focus primarily on participation and which are not prevalent in the field of mental illness requires attention. We hypothesize the synonymous use of terms may be partially explained by the fact that participation has not been clearly defined alongside related concepts of social functioning, quality of life and well-being. This also reflects that the field of rehabilitation and medicine has claimed participation within a service-oriented and needs-based perspective.

In LMICs, there is a paucity of context-validated scales to look at lives of persons with mental illness. Further, there is a need for participation scales that focus on empowerment as well as collective capabilities. This also means that participation measurement strongly needs to be grounded within a rights-based perspective like the United Nation Convention on the Rights of Persons with Disabilities.

## Additional file

Additional file 1:
**Flow Diagram.** (DOC 55 kb)
