# Supplementary material for: Measuring participation for persons with mental illness: A systematic review assessing relevance of existing scales for low and middle income countries
Source: BMC Psychol. 2015 Oct 14;3:36. doi: 10.1186/s40359-015-0093-0 (PMC4607168; doi:10.1186/s40359-015-0093-0)
Supplement: Additional file 1: — Flow Diagram. (DOC 55 kb) [file 40359_2015_93_MOESM1_ESM.doc]

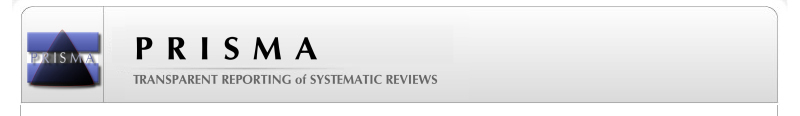
**PRISMA 2009 Flow Diagram**

**Screening**

**Included**

**Eligibility**

**Identification**

Records identified through database searching
(n = 191)

Records after duplicates removed
(n = 143)

Records screened
(n = 143)

Records excluded
(n = 58)

Full-text articles assessed for eligibility
(n = 85)

Scales identified using keywording tool
(n = 48)

Scales assessed based on established criteria

(n = 34)

Scales excluded, due to not being available

(n = 14)

Scales included in systematic review

(n = 5)
